# Dokdonella aquatica sp. nov., isolated from freshwater

**DOI:** 10.1099/ijsem.0.007172

**Published:** 2026-05-15

**Authors:** Jae Kyeong Lee, Chae Woo Lim, Chae Yeong Moon, Sung Chul Lee, Che Ok Jeon

**Affiliations:** 1 Department of Life Science, Chung-Ang University, Seoul 06974, Republic of Korea

**Keywords:** *Dokdonella aquatica*, freshwater, new taxa, *Pseudomonadota*

## Abstract

A Gram-stain-negative, strictly aerobic, non-motile, non-flagellated and rod-shaped bacterium, strain MW10^T^, was isolated from freshwater in South Korea. The strain exhibited catalase-negative and oxidase-positive activities and grew at 15–35 °C (optimum, 25 °C), pH 6.0–9.0 (optimum, 7.0) and in 0–3.0% (w/v) NaCl (optimum, 0%). Ubiquinone-8 was the sole respiratory quinone, and the major cellular fatty acids (>10%) were iso-C_15 : 0_, iso-C_16 : 0_ and summed feature 9 (iso-C_17 : 1_* ω9*c and/or C_16 : 0_ 10-methyl). Polar lipids comprised phosphatidylethanolamine, phosphatidylglycerol, diphosphatidylglycerol, an unidentified aminophospholipid and an unidentified glycolipid. The genome of strain MW10^T^ was 4831 kb in size with a DNA G+C content of 69.0 mol%. Strain MW10^T^ was most closely related to *Dokdonella immobilis* LM 2-5^T^ and *Dokdonella koreensis* DS-123^T^ with 16S rRNA gene sequence similarities of 97.1% and 96.0%, respectively. Phylogenetic analyses based on 16S rRNA gene and 120 marker protein sequences placed strain MW10^T^ within the genus *Dokdonella* as a distinct lineage. Average nucleotide identity and digital DNA–DNA hybridization values between strain MW10^T^ and related type strains were ≤76.6% and ≤21.1%, respectively. Collectively, phylogenetic, genomic, phenotypic and chemotaxonomic evidence support the conclusion that strain MW10^T^ represents a novel species of the genus *Dokdonella*, for which the name *Dokdonella aquatica* sp. nov. is proposed. The type strain is MW10^T^ (=KACC 23687^T^=JCM 36943^T^).

## Introduction

The genus *Dokdonella*, belonging to the family *Rhodanobacteraceae* within the phylum *Pseudomonadota*, was established in 2006 with *Dokdonella koreensis* as the type species, originally isolated from soil [1]. The genus has since been emended through several taxonomic revisions [2,3] and currently comprises six validly published species as of 18 January 2026 (https://lpsn.dsmz.de/genus/dokdonella). Members of *Dokdonella* have been isolated predominantly from terrestrial environments, including soil from ginseng fields [2], activated sludge [3], potting soil [4], soil [1,5] and batch reactors treating triphenylmethane dye effluents [6].

Species of this genus are generally characterized as Gram-stain-negative, rod-shaped bacteria that are typically oxidase-positive and catalase-variable [1,6]. Chemotaxonomically, they possess ubiquinone-8 (Q-8) as the major respiratory quinone, branched-chain fatty acids such as iso-C_15 : 0_, iso-C_16 : 0_, iso-C_17 : 0_ and C_17 : 1_* ω9*c as major cellular fatty acids, and phosphatidylethanolamine (PE), phosphatidylglycerol (PG) and diphosphatidylglycerol (DPG) as the principal polar lipids. As part of a Korean government-supported microbial resource collection programme, we have isolated and characterized bacteria from diverse environmental samples [7,10]. In the course of this work, a putative novel strain affiliated with the genus *Dokdonella* was isolated from freshwater, and its taxonomic position was investigated using a polyphasic approach.

## Strain isolation

Strain MW10^T^ was isolated from freshwater collected from a stream in Siheung City, Republic of Korea (37° 23′ 7″ N 126° 50′ 21″ E), as previously described [7]. Briefly, the sample was serially diluted in 0.9% (w/v) saline, and 100 µl aliquots of each dilution were spread onto Reasoner’s 2A (R2A) agar (MBcell, South Korea). Plates were incubated aerobically at 25 °C for 2 days. Colonies grown on R2A agar were resuspended in 100 µl of 5% (w/v) Chelex-100 solution (Bio-Rad) and boiled for 10 min to prepare crude genomic DNA lysates. The 16S rRNA gene was amplified using universal primers 27F (5′-AGA GTT TGA TCM TGG CTC AG-3′) and 1492R (5′-TAC GGY TAC CTT GTT ACG ACT T-3′) [11]. PCR amplicons were digested with HaeIII and HhaI and separated by electrophoresis on 2% agarose gels. Representative amplicons with distinct restriction fragment patterns were sequenced using the universal primer 340F (5′-CCTACGGGAGGCAGCAG-3′) [11]. Partial 16S rRNA gene sequences were compared with those of type strains using the EzBioCloud server (https://www.ezbiocloud.net/identify) [12]. Based on these comparisons, strain MW10^T^, putatively affiliated with the genus *Dokdonella*, was selected for further taxonomic characterization.

The strain was routinely cultivated on R2A agar at 25 °C for 2 days and preserved at –80 °C in R2A broth supplemented with 15% (v/v) glycerol. Type strains *Dokdonella immobilis* JCM 15763^T^ and *D. koreensis* KCTC 12396^T^, which showed the highest 16S rRNA gene sequence similarity to strain MW10^T^ and clustered most closely with it in phylogenetic analyses, were obtained from their respective culture collections and used as reference strains for phenotypic, chemotaxonomic and fatty acid profile comparisons.

## Phylogeny based on 16S rRNA gene sequence

The 16S rRNA gene amplicon of strain MW10^T^, initially amplified using primers 27F and 1492R, was further sequenced with the universal primers 518R (5′-ATT ACC GCG GCT GCT GG-3′) and 805F (5′-GAT TAG ATAC CCT GGT AGT C-3′) [11]. Sequences obtained using primers 340F, 518R and 805F were assembled to generate a nearly complete 16S rRNA gene sequence (1,486 nt) for strain MW10^T^. Sequence similarities between strain MW10^T^ and closely related type strains were calculated using the EzBioCloud server (http://www.ezbiocloud.net/identify) [12]. Multiple sequence alignment of the 16S rRNA gene sequences of strain MW10^T^ and related type strains was performed using Infernal (version 1.1.4) [13]. Phylogenetic relationships were inferred using neighbour-joining (NJ), maximum-parsimony (MP) and maximum-likelihood (ML) methods implemented in mega11 [14], with bootstrap support assessed from 1,000 replicates. The Kimura two-parameter model, nearest-neighbour interchange heuristic search and subtree-pruning-and-regrafting algorithm were applied for NJ, ML and MP tree construction, respectively.

**Fig. 1.**
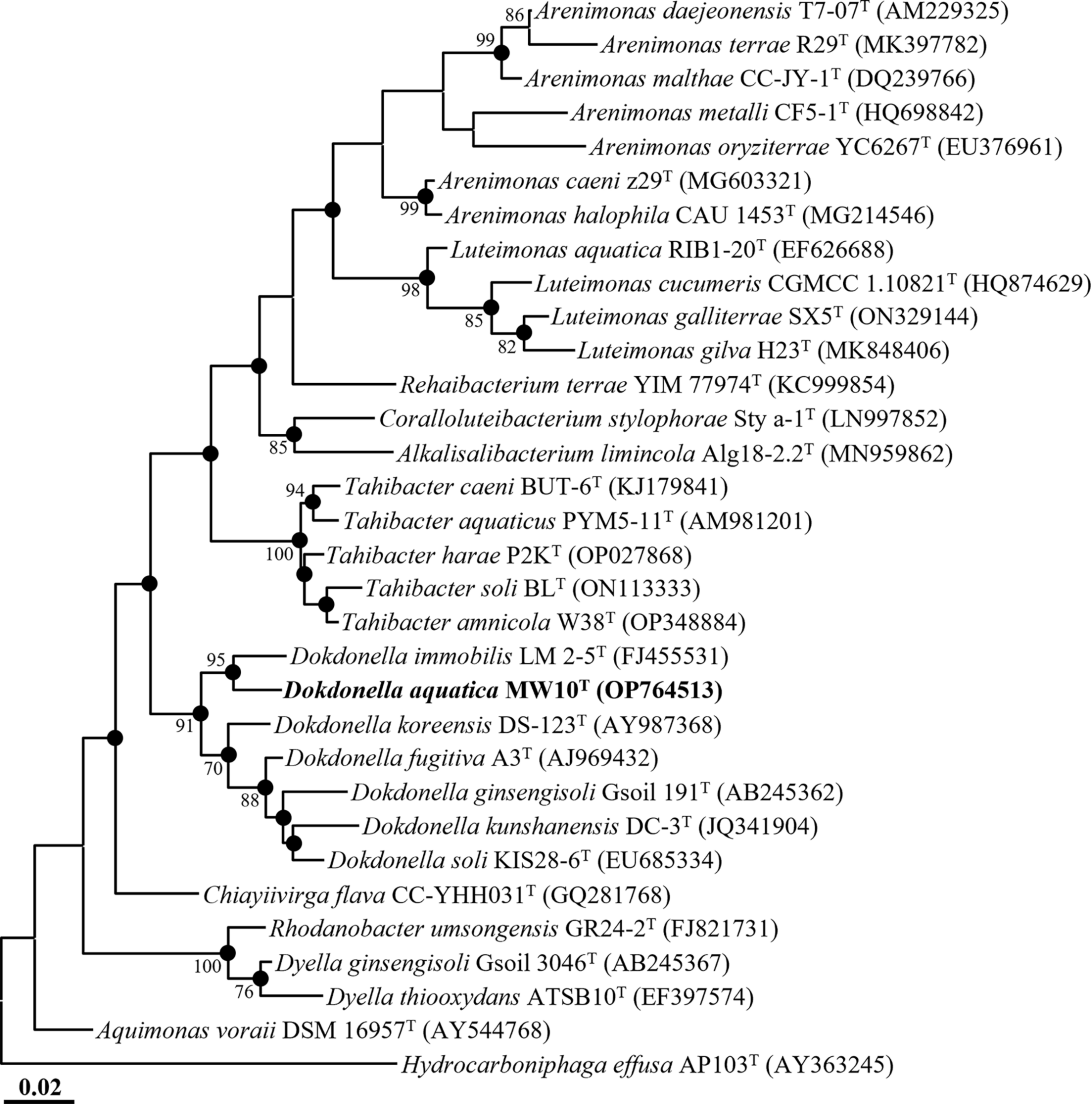
ML phylogenetic tree showing the relationships of strain MW10^T^ and closely related type strains based on 16S rRNA gene sequences. Bootstrap values (>70%) based on 1,000 replicates are shown at the nodes. Filled circles (●) indicate nodes also recovered in the NJ and MP trees. *Hydrocarboniphaga effusa* AP103^T^ (AY363245) was used as the outgroup. The scale bar represents 0.02 substitutions per nucleotide position.

Comparative analysis of 16S rRNA gene sequences showed that strain MW10^T^ was most closely related to *D. immobilis* LM2-5^T^ and *Dokdonella ginsengisoli* Gsoil 191^T^, with sequence similarities of 97.1% and 96.4%, respectively. These values are below the species delineation threshold of 98.7% [15], suggesting that strain MW10^T^ may represent a distinct species. Phylogenetic analysis using 16S rRNA gene sequences based on the ML method showed that strain MW10^T^ formed a phylogenetic lineage with *D. immobilis* LM2-5^T^ within the genus *Dokdonella*, with a high bootstrap value (95%) (Fig. 1). NJ and MP analyses also yielded congruent topologies with high bootstrap supports of 98% and 91%, respectively (Fig. S1, available in the online Supplementary Material). Collectively, these comparative and phylogenetic analyses based on 16S rRNA gene sequences support the classification of strain MW10^T^ as a novel species within the genus *Dokdonella*.

## Ecological distribution analysis

To assess the ecological distribution of strain MW10^T^, its 16S rRNA gene sequence was compared with 500,048 metagenomic 16S rRNA amplicon datasets from diverse environments, including freshwater, soil, seawater, marine and freshwater sediments, plants, algae, fish, biofilms, food and human and animal gut samples, using the Integrated Microbial Next-Generation Sequencing (IMNGS) platform [16] at a 99.0% sequence similarity threshold, which was selected to provide high taxonomic resolution and minimize overlap with closely related *Dokdonella* species, as the highest 16S rRNA gene sequence similarity among validly described members of the genus is 98.1%.

IMNGS analysis revealed that bacteria closely related to strain MW10^T^ are widely distributed across diverse environments, including sludge metagenomes, wastewater, compost, freshwater, aerosol and insect- and plant-associated habitats (Table S1). These sequences were most frequently and abundantly detected in water-associated environments such as sludge metagenomes, wastewater and freshwater, consistent with the isolation of strain MW10^T^ from freshwater. In contrast, related sequences were also detected in various insect-associated metagenomes, including *Bembidion* species, *Rhipicephalus sanguineus*, *Calliphora* species and beetle metagenomes, but with low average relative abundance (ARA) values. Given that strain MW10ᵀ is strictly aerobic, insect-associated environments are unlikely to represent its primary ecological niche. Sequences closely related to strain MW10^T^ were additionally detected in plant-, fish-, *Gorilla gorilla*-, eye- and aerosol-associated metagenomes at very low prevalence or ARA values. Collectively, these findings indicate that freshwater and other water-associated environments constitute the primary habitats of strain MW10^T^, whereas its presence in insect-, plant- and other host- or environment-associated metagenomes likely reflects indirect associations via environmental exposure or trophic transfer rather than stable colonization.

## Whole-genome sequencing and genome-based phylogeny

Genomic DNA of strain MW10^T^ was extracted from cells grown in R2A broth using the Wizard Genomic DNA Purification Kit (Promega, USA), following the manufacturer’s protocol. Whole-genome sequencing was performed using a hybrid approach combining the Oxford Nanopore MinION platform (Oxford Nanopore Technologies, UK) and the Illumina NovaSeq 6000 platform (Illumina, USA) with 151-bp paired-end reads. Nanopore long reads were *de novo* assembled using Flye v2.9.1 [17]. The resulting assembly was polished with Illumina short-read data using Pilon v1.24 [18], with iterative rounds of polishing performed until no further corrections were detected. Genome quality was assessed using CheckM2 (version 1.0.2) based on estimates of completeness and contamination [19]. Phylogenomic relationships between strain MW10^T^ and closely related type strains were inferred using the Genome Taxonomy Database Toolkit (GTDB-Tk) based on concatenated amino acid sequences of 120 ubiquitous single-copy marker genes (bac120 marker set) [20]. The resulting ML phylogenomic tree was constructed in mega11 with bootstrap support calculated from 1,000 replicates. Genome relatedness between strain MW10^T^ and related type strains was further evaluated by calculating average nucleotide identity (ANI) and digital DNA–DNA hybridization (dDDH) values using the Orthologous ANI Tool on the EzBioCloud server (https://www.ezbiocloud.net/tools/orthoani) [21] and the Genome-to-Genome Distance Calculator (version 2.1; https://ggdc.dsmz.de/distcalc2.php) [22], respectively.

A complete genome sequence of strain MW10^T^, ~4,831 kb in size, was obtained by *de novo* assembly of Oxford Nanopore MinION reads (~61× coverage; National Center for Biotechnology Information (NCBI) SRA accession no. SRR38108275), followed by polishing with Illumina reads (~975× coverage; NCBI SRA accession no. SRR38108274), exceeding the generally recommended ≥50× coverage for taxonomic studies [15]. The 16S rRNA gene sequence identified in the genome was identical to that obtained by PCR-based sequencing. Genome quality assessment using CheckM2 confirmed a high-quality assembly, with 100% completeness and a contamination level of 0.05%, meeting the accepted criteria for high-quality genomes (completeness ≥90%, contamination ≤10%) [15].

**Fig. 2.**
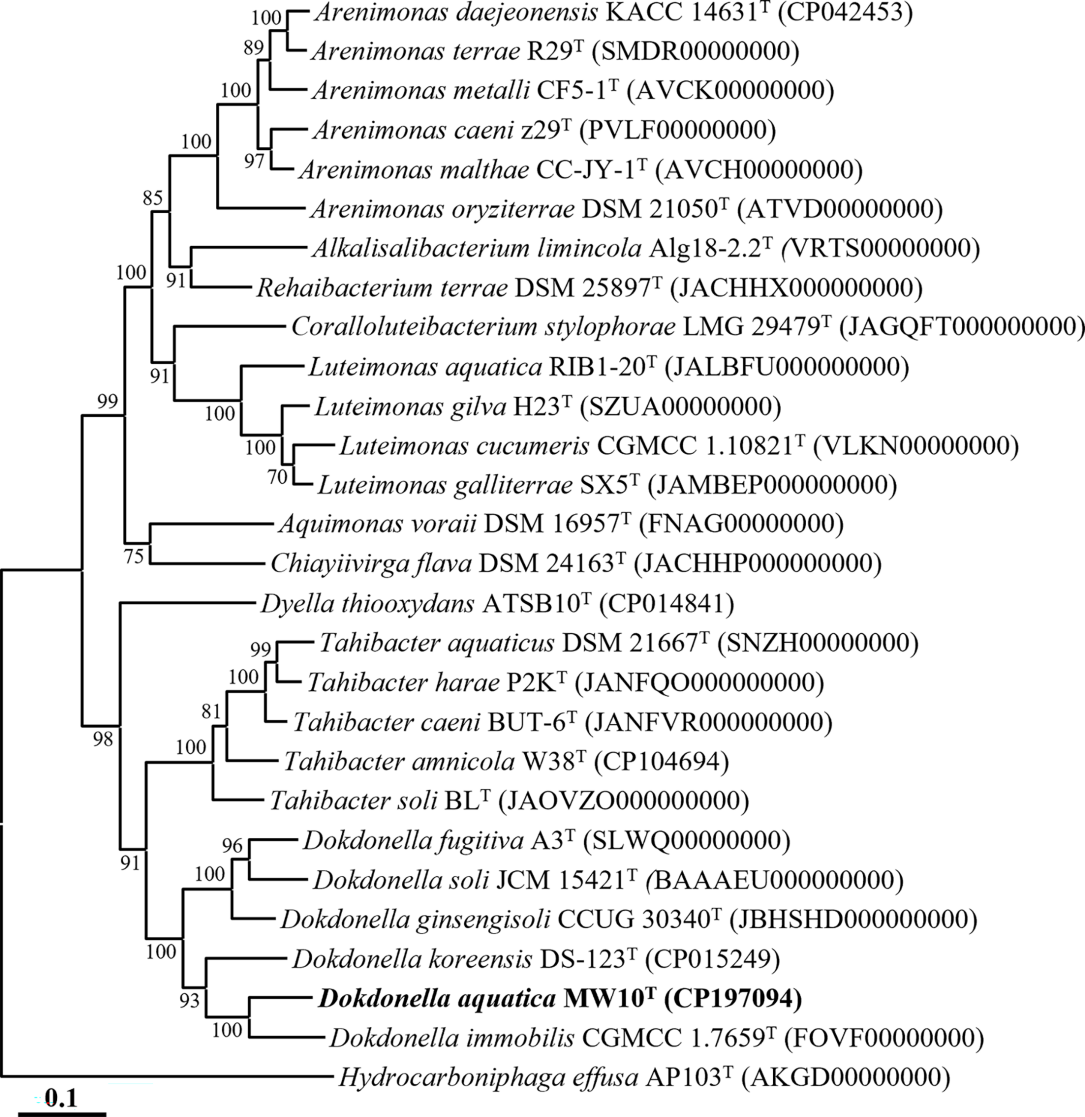
Phylogenomic tree showing the relationships of strain MW10^T^ and closely related type strains based on concatenated protein sequences of 120 bacterial marker genes (bac120) generated using GTDB-Tk. Bootstrap values (>70%) based on 1,000 replicates are shown at the nodes. *Hydrocarboniphaga effusa* AP103^T^ (AKGD00000000) was used as the outgroup. The scale bar represents 0.1 substitutions per amino acid position.

Genome-based phylogenomic analysis showed that strain MW10^T^ formed a well-supported lineage with *D. immobilis* CGMCC 1.7659^T^ within the genus *Dokdonella* (Fig. 2), consistent with the placement inferred from 16S rRNA gene sequence-based analyses (Figs1  2). The ANI and dDDH values between strain MW10^T^ and closely related *Dokdonella* type strains were ≤76.6% and ≤21.1%, respectively (Table S2), which are well below the established thresholds for species delineation (ANI≈95–96%; dDDH 70%) [15]. Collectively, these phylogenomic and genome-relatedness analyses support the conclusion that strain MW10^T^ represents a novel species within the genus *Dokdonella*.

## Genomic features

The whole-genome sequence of strain MW10^T^ was deposited in the GenBank database and annotated using the NCBI Prokaryotic Genome Annotation Pipeline. The genome comprises 4,283 genes, including 4,223 protein-coding sequences, two complete rRNA operons (16S, 23S and 5S), 50 tRNA genes representing all 20 amino acids and 4 non-coding RNA genes (Table 1). The genomic DNA G+C content of strain MW10^T^, calculated from the complete genome sequence, is 69.0 mol%, which is consistent with values reported for other *Dokdonella* species [1,6]. Comparative analysis of general genomic features showed that strain MW10^T^ is comparable to closely related type strains of the genus *Dokdonella* (Table 1).

**Table 1.** General genomic features of strain MW10^T^ and closely related type strains of the genus *Dokdonella* Taxa: 1, strain MW10^T^ (CP197094); 2, *D. immobilis* CGMCC 1.7659^T^ (NZ_FOVF00000000); 3, *D. koreensis* DS-123^T^ (NZ_CP015249).

Feature*	1	2	3
Genome status^†^ (no. of contigs)	C (1)	D (82)	C (1)
Genome size (kb)	4831	4635	4446
G+C content (%)	69.0	65.0	70.5
No. of total genes	4283	3970	3653
No. of protein-coding genes	4223	3868	3568
No. of total RNA genes	60	53	57
No. of tRNA genes	50	45	46
No. of rRNA (16S, 23S, 5S) operons	2	1	2
No. of non-coding RNA genes	4	5	1
No. of pseudogenes	33	49	28

*The genomic features were obtained from the NCBI prokaryotic genome annotation pipeline (www.ncbi.nlm.nih.gov/genome/annotation_prok/).

†C, complete; D, draft.

## Growth, morphology and physiological properties

Growth of strain MW10^T^ was examined on marine agar (MA), R2A agar, Luria–Bertani (LB) agar, tryptic soy agar (TSA), and nutrient agar (NA) (all from MBcell) after incubation at 25 °C for 2 days. Temperature tolerance was assessed on R2A agar at 10–45 °C (5 °C intervals), and pH tolerance was tested in R2A broth adjusted to pH 4.0–10.0 (1.0-unit intervals) and incubated at 25 °C for 2 days. Media were buffered with sodium citrate (pH 4.0–5.0), sodium phosphate (pH 6.0–8.0) or sodium carbonate–bicarbonate (pH 9.0–10.0), with final pH adjustment after autoclaving when necessary. NaCl tolerance was evaluated in R2A broth supplemented with 0–5.0% (w/v) NaCl at 1.0% intervals, incubated at 25 °C for 2 days.

Cell shape and motility were examined by phase-contrast microscopy (Axio Scope.A1, Zeiss, Germany). For transmission electron microscopy, cells grown on R2A agar at 25 °C for 2 days were negatively stained with 2% (w/v) uranyl acetate and observed using a JEM-1010 microscope (JEOL, Japan). Gliding motility was assessed on R2A semi-solid agar (0.3%, w/v). Gram staining was performed using a commercial kit (bioMérieux, France). Catalase and oxidase activities were tested using 3% (v/v) hydrogen peroxide and 1% (w/v) tetramethyl-*p*-phenylenediamine, respectively [23]. Anaerobic growth was evaluated on R2A agar after 21 days at 25 °C using the GasPak Plus system (BBL, USA). Phenotypic characteristics of strain MW10^T^ were compared with those of reference type strains under identical conditions at their respective optimal growth temperatures. Hydrolysis of tyrosine, casein, aesculin, starch, Tween 20 and Tween 80 was assessed on R2A agar as previously described [23], and additional biochemical characteristics were determined using API 20NE kits (bioMérieux) according to the manufacturer’s instructions.

Strain MW10^T^ grew well on R2A agar; showed slow growth on TSA, NA, and LB agar; and did not grow on MA. Cells were Gram-stain-negative, non-motile rods lacking flagella, measuring 0.4–0.5 µm in width and 2.2–2.4 µm in length (Fig. S2). The strain did not grow under anaerobic conditions, indicating an obligately aerobic lifestyle (Table 2).

**Table 2.** Differential characteristics between strain MW10^T^ and its closely related type strains of the genus *Dokdonella* Taxa: 1, strain MW10^T^ (this study); 2, *D. immobilis* JCM 15763^T^ [6]; 3, *D. koreensis* KCTC 12396^T^ [1]. All strains form yellow colonies and are strictly aerobic and rod-shaped bacteria. All strains are uniformly positive for oxidase and *β*-glucosidase* activities, hydrolysis* of casein, Tween 80, tyrosine, aesculin and gelatin and assimilation* of l-arabinose, *N-*acetylglucosamine, potassium gluconate, adipic acid and malic acid. All strains are negative for Gram-staining, flagellar motility, gliding motility, indole production*, urease, arginine dihydrolase and *β*-galactosidase activities*; hydrolysis* of Tween 20 and starch, glucose fermentation*; and assimilation* of d-mannose, capric acid and phenylacetic acid. Symbols: +, positive; –, negative; na, not available.

Characteristic	1	2	3
Isolation source	Fresh water	Sludge	Soil
Growth range of			
Temperature (°C, optimum)	15–35 (25)	16–37 (25)	10–39 (30)
NaCl (%, optimum)	0–3.0 (0)	0–2.0 (na)	0–3.0 (0)
pH (optimum)	6.0–9.0 (7.0)	5.0–8.5 (6.5–7.0)	6.5–7.5 (6.5)
Catalase	–	–	+
Nitrate reduction to nitrite*	+	–	+
Assimilation* of			
Trisodium citrate	+	–	–
Potassium citrate, d-maltose	+	–	+
d-Glucose, d-mannitol	–	–	+
Major fatty acids (>10%)	iso-C_15 : 0_, iso-C_16 : 0_, summed feature 9^†^	iso-C_15 : 0_, iso-C_16 : 0_, summed feature 9^†^	iso-C_11 : 0_, iso-C_16 : 0_, summed feature 9^†^

*These data were obtained from this study under the same conditions.

†Summed feature 9: iso-C_17 : 1_* ω*9*c* and/or C_16 : 0_ 10-methyl.

Strain MW10^T^ shared several phenotypic characteristics with reference strains of the genus *Dokdonella*, including rod-shaped morphology, Gram-stain negativity, the absence of indole production and glucose fermentation, oxidase and *β*-glucosidase activities; hydrolysis of casein, Tween 80, tyrosine, aesculin and gelatin; and assimilation of l-arabinose, *N*-acetylglucosamine, potassium gluconate, adipic acid, malic acid, d-mannose, capric acid and phenylacetic acid (Table 2). However, it could be differentiated from closely related *Dokdonella* species by differences in growth temperature, salinity and pH ranges, catalase activity, nitrate reduction to nitrite and assimilation of several substrates, including trisodium citrate, d-maltose, d-glucose and d-mannitol (Table 2).

## Chemotaxonomic characteristics

Respiratory isoprenoid quinones were extracted from strain MW10^T^ after cultivation in R2A broth at 25 °C for 2 days and analysed using an HPLC system (LC-20A, Shimadzu, Japan) equipped with a reversed-phase column (250×4.6 mm; Kromasil, Akzo Nobel, Netherlands) and a diode-array detector (SPD-M20A; Shimadzu), following the method of Minnikin *et al*. [24]. A methanol–isopropanol (2 : 1, v/v) mixture was used as the mobile phase at a flow rate of 1 ml min^−1^. For cellular fatty acid analysis, strain MW10^T^ and two reference strains were cultivated aerobically in R2A broth at their respective optimal temperatures and harvested during the exponential growth phase (OD_600_=0.7–0.8). Fatty acid methyl esters were prepared according to the standard MIDI protocol (Sherlock Microbial Identification System, version 6.2B), including saponification, methylation and extraction, and analysed using a gas chromatograph (HP 6890; Hewlett-Packard, USA). Fatty acids were identified using the RTSBA6 database (Sherlock version 6.0B) [25]. Polar lipids were extracted from exponential-phase cells of strain MW10^T^ and separated by two-dimensional TLC as described by Minnikin *et al*. [26]. Lipid classes were detected using 10% ethanolic molybdophosphoric acid for total lipids, ninhydrin for aminolipids, Dittmer–Lester reagent for phospholipids and *α*-naphthol/sulfuric acid for glycolipids. The presence of PE, PG and DPG was confirmed using authentic standards (Sigma-Aldrich, USA).

Q-8 was identified as the sole respiratory isoprenoid quinone in strain MW10^T^, consistent with other members of the genus *Dokdonella* [1,6]. The major cellular fatty acids (>10% of total fatty acids) were iso-C_15 : 0_ (23.1%), iso-C_16 : 0_ (21.0%) and summed feature 9 (comprising iso-C_17 : 1_* ω9*c and/or C_16 : 0_ 10-methyl, 23.8%) (Table S3). Although the overall fatty acid profile of strain MW10^T^ was similar to those of closely related *Dokdonella* type strains, differences were observed in the relative abundance of specific components. Notably, iso-C_14 : 0_ and anteiso-C_17 : 0_ were detected in strain MW10^T^ but were absent from *D. koreensis* KCTC 12396^T^ and *D. immobilis* JCM 15763^T^, respectively. PE, PG and DPG were detected in strain MW10^T^ as polar lipids (Fig. S3), consistent with other *Dokdonella* species [1,6]. In addition, an unidentified aminophospholipid and an unidentified glycolipid were detected in strain MW10^T^.

## Taxonomic conclusion

Collectively, phylogenetic, genomic, phenotypic, physiological and chemotaxonomic evidence support the conclusion that strain MW10^T^ represents a novel species within the genus *Dokdonella*. Accordingly, the name *Dokdonella aquatica* sp. nov. is proposed for strain MW10^T^.

## Description of *Dokdonella aquatica* sp. nov.

*Dokdonella aquatica* (a.qua’ti.ca. L. fem. adj. *aquatica*, living in water, aquatic).

Colonies on R2A agar are circular, convex and yellow. Cells are Gram-stain-negative, strictly aerobic, non-motile rods lacking flagella and gliding motility. Growth occurs at 15–35 °C (optimum, 25 °C), pH 6.0–9.0 (optimum, pH 7.0) and in the presence of 0–3.0% (w/v) NaCl (optimum, 0%). Cells are catalase-negative and oxidase-positive, and nitrate is reduced to nitrite. Indole production and glucose fermentation are negative. Casein, Tween 80, tyrosine, aesculin and gelatin are hydrolysed, whereas Tween 20 and starch are not. *β*-Glucosidase activity is positive, while urease, arginine dihydrolase and *β*-galactosidase activities are negative. l-Arabinose, *N*-acetylglucosamine, potassium gluconate, adipic acid, malic acid, trisodium citrate, potassium citrate and d-maltose are assimilated, whereas d-glucose, d-mannose, capric acid, phenylacetic acid and d-mannitol are not. The sole respiratory quinone is Q-8. Polar lipids comprise PG, DPG, PE, an unidentified aminophospholipid and an unidentified glycolipid. Major cellular fatty acids (>10%) are iso-C_15 : 0_, iso-C_16 : 0_ and summed feature 9 (iso-C_17 : 1_* ω*9*c* and/or C_16 : 0_ 10-methyl).

The type strain is MW10^T^ (=KACC 23687^T^=JCM 36943^T^), isolated from fresh water collected in South Korea. The genome size and DNA G+C content of the type strain are 4831 kb and 69.0 mol% (calculated from the whole-genome sequence), respectively. The GenBank accession numbers of the 16S rRNA gene and genome sequences of strain MW10^T^ are OP764513 and CP197094, respectively.

## Supplementary material

10.1099/ijsem.0.007172 Supplementary Material 1.

## Abbreviations

ANI: average nucleotide identity
ARA: average relative abundance
dDDH: digital DNA–DNA hybridization
DPG: diphosphatidylglycerol
GTDB-Tk: Genome Taxonomy Database Toolkit
IMNGS: Integrated Microbial Next-Generation Sequencing
LB: Luria–Bertani
MA: marine agar
ML: maximum-likelihood
MP: maximum-parsimony
NA: nutrient agar
NJ: neighbour-joining
PE: phosphatidylethanolamine
PG: phosphatidylglycerol
Q-8: ubiquinone-8
R2A: Reasoner’s 2A
TSA: tryptic soy agar

## References

[1] Yoon JH (2006). *Dokdonella koreensis* gen. nov., sp. nov., isolated from soil. Int J Syst Evol Microbiol.

[2] Ten LN, Jung H-M, Im W-T, Oh HW, Yang D-C (2009). *Dokdonella ginsengisoli* sp. nov., isolated from soil from a ginseng field, and emended description of the genus *Dokdonella*. Int J Syst Evol Microbiol.

[3] Li Y, Zhang J, Chen Q, Yang G, Cai S (2013). *Dokdonella kunshanensis* sp. nov., isolated from activated sludge, and emended description of the genus *Dokdonella*. Int J Syst Evol Microbiol.

[4] Cunha S, Tiago I, Luísa Pires A, da Costa MS, Veríssimo A (2006). *Dokdonella fugitiva* sp. nov., a Gammaproteobacterium isolated from potting soil. Syst Appl Microbiol.

[5] Yoo SH, Weon HY, Anandham R, Kim BY, Hong SB (2009). A gammaproteobacterium isolated from soil. Int J Syst Evol Microbiol.

[6] Liu Y, Jin J-H, Liu H-C, Liu Z-P (2013). *Dokdonella immobilis* sp. nov., isolated from a batch reactor for the treatment of triphenylmethane dye effluent. Int J Syst Evol Microbiol.

[7] Yu YU, Lee SH, Jeon CO, Kim KH (2026). *Methylobacterium synurae* sp. nov., isolated from a freshwater alga *Synura petersenii*. Int J Syst Evol Microbiol.

[8] Baek JH, Han DM, Choi DG, Moon CY, Lee JK (2025). *Staphylococcus parequorum* sp. nov. and *Staphylococcus halotolerans* sp. nov., isolated from traditional Korean soybean foods. J Microbiol.

[9] Baek JH, Han DM, Kim JM, Jia B, Jung JY (2023). *Tahibacter soli* sp. nov., isolated from soil and *Tahibacter amnicola* sp. nov., isolated from freshwater. Int J Syst Evol Microbiol.

[10] Kim JM, Choi BJ, Bayburt H, Han DM, Jeon CO (2025). *Aquimarina rhodophyticola* sp. nov. and *Aquimarina besae* sp. nov., isolated from marine red algae. Curr Microbiol.

[11] Lane DJ. (2022). 6S/23S rRNA Sequencing. 1991. Nucleic Acid Techniques in Bacterial Systematics.

[12] Yoon S-H, Ha S-M, Kwon S, Lim J, Kim Y (2017). Introducing EzBioCloud: a taxonomically united database of 16S rRNA gene sequences and whole-genome assemblies. Int J Syst Evol Microbiol.

[13] Nawrocki EP, Eddy SR (2013). Infernal 1.1: 100-fold faster RNA homology searches. Bioinformatics.

[14] Tamura K, Stecher G, Kumar S (2021). MEGA11: Molecular evolutionary genetics analysis version 11. Mol Biol Evol.

[15] Riesco R, Trujillo ME (2024). Update on the proposed minimal standards for the use of genome data for the taxonomy of prokaryotes. Int J Syst Evol Microbiol.

[16] Lagkouvardos I, Joseph D, Kapfhammer M, Giritli S, Horn M (2016). IMNGS: a comprehensive open resource of processed 16S rRNA microbial profiles for ecology and diversity studies. Sci Rep.

[17] Kolmogorov M, Yuan J, Lin Y, Pevzner PA (2019). Assembly of long, error-prone reads using repeat graphs. Nat Biotechnol.

[18] Walker BJ, Abeel T, Shea T, Priest M, Abouelliel A (2014). Pilon: an integrated tool for comprehensive microbial variant detection and genome assembly improvement. PLoS One.

[19] Chklovski A, Parks DH, Woodcroft BJ, Tyson GW (2023). CheckM2: a rapid, scalable and accurate tool for assessing microbial genome quality using machine learning. Nat Methods.

[20] Chaumeil PA, Mussig AJ, Hugenholtz P, Parks DH (2020). GTDB-Tk: a toolkit to classify genomes with the genome taxonomy database. Bioinformatics.

[21] Lee I, Ouk Kim Y, Park S-C, Chun J (2016). OrthoANI: an improved algorithm and software for calculating average nucleotide identity. Int J Syst Evol Microbiol.

[22] Meier-Kolthoff JP, Auch AF, Klenk HP, Göker M (2013). Genome sequence-based species delimitation with confidence intervals and improved distance functions. BMC Bioinformatics.

[23] Smibert RM, Krieg NR, Gerhardt P (1994). Methods for General and Molecular Bacteriology.

[24] Minnikin DE, O’Donnell AG, Goodfellow M, Alderson G, Athalye M (1984). An integrated procedure for the extraction of bacterial isoprenoid quinones and polar lipids. J Microbiol Methods.

[25] Sasser M (1990). MIDI Technical Note.

[26] Minnikin DE, Patel PV, Alshamaony L, Goodfellow M (1977). Polar lipid composition in the classification of nocardia and related bacteria. Int J Syst Bacteriol.

