# Supplementary material for: Dokdonella aquatica sp. nov., isolated from freshwater
Source: Int J Syst Evol Microbiol. 2026 May 15;76(5):007172. doi: 10.1099/ijsem.0.007172 (PMC13178995; doi:10.1099/ijsem.0.007172)
Supplement: Supplementary Material 1. [file ijsem-76-07172-s001.pdf]

Supplementary Information

Fig. S1. Neighbor-joining (NJ; a) and maximum-parsimony (MP; b) phylogenetic trees showing the relationships of strain MW10^{T} and closely related strains based on 16S rRNA gene sequences. Bootstrap values (>70%) based on 1000 replicates are shown at the nodes. *Hydrocarboniphaga effusa* AP103^{T} (AY363245) was used as the outgroup. Scale bars in the NJ and MP trees represent nucleotide substitutions per site and the total number of substitutions across the full sequence, respectively.

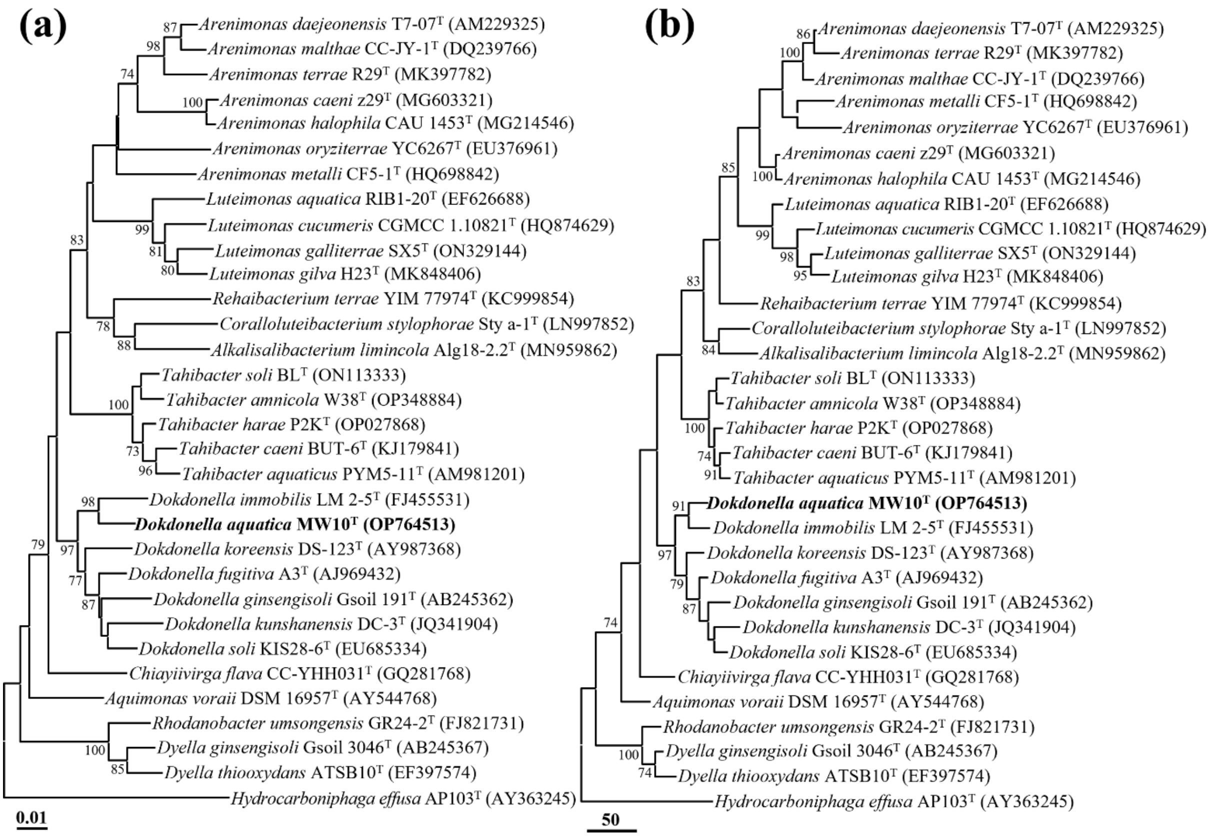

Fig. S2. Transmission electron micrograph showing the cellular morphology of strain MW10^T, negatively stained with 2% (w/v) uranyl acetate after cultivation on R2A agar at 25°C for 2 days. The scale bar represents 1 μm.

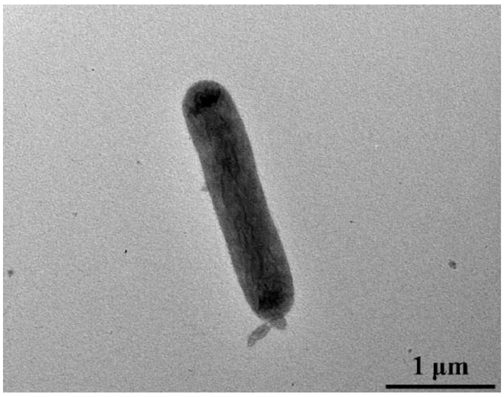

Fig. S3. Two-dimensional thin-layer chromatograms (TLC) showing the polar lipid of strain MW10^T. The solvent systems used were (I) chloroform-methanol-water (65:25:4, v/v/v) and (II) chloroform-acetic acid-methanol-water (80:15:12:4, v/v/v/v). TLC plates were sprayed with 10% ethanolic molybdophosphoric acid (a), ninhydrin (b), Dittmer-Lester (c), and *α*-naphthol/sulfuric acid (d) to detect total polar lipids, aminolipids, phospholipids, and glycolipids, respectively. Abbreviations: PE, phosphatidylethanolamine; PG, phosphatidylglycerol; DPG, diphosphatidylglycerol; APL, aminophospholipid; GL, glycolipid.

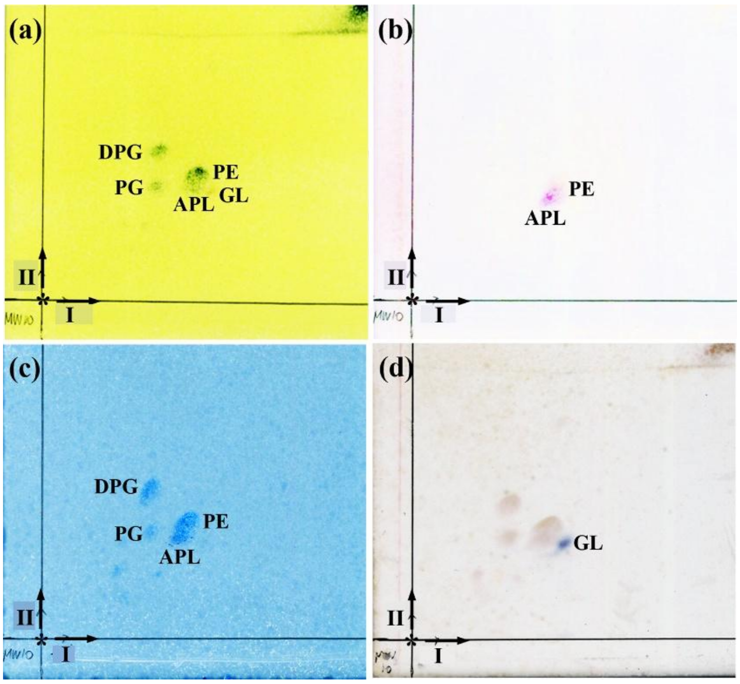

Table S1. Potential ecological distributions of strain MW10^T assessed by comparing its 16S rRNA gene sequence with metagenomic 16S rRNA amplicon datasets using the Integrated Microbial Next-Generation Sequencing (IMNGS) platform at a 99.0% sequence similarity threshold. "Matched no. (prevalence)" indicates the number (percentage) of datasets containing sequences matching the 16S rRNA gene of strain MW10^T, and "ARA" denotes the average relative abundance of these sequences within the datasets. Prevalence and ARA values below 1.0% and 0.0001%, respectively, are not shown.

**Habitat**	**No. of datasets**	**Matched no. (prevalence, %)**	**ARA (%)**
Sludge metagenome	1924	155 (8.1)	0.0216
Anaerobic digester metagenome	1379	70 (5.1)	0.0216
Bioreactor sludge metagenome	542	27 (5.0)	0.0061
Wastewater metagenome	2738	76 (2.8)	0.0054
Activated sludge metagenome	2846	42 (1.5)	0.0087
Compost metagenome	383	7 (1.8)	0.0575
Riverine metagenome (freshwater)	349	8 (2.3)	0.0152
Bembidion decorum (insect)	9	1 (11.1)	0.0027
Paederidus rubrothoracicus (insect)	9	1 (11.1)	0.0011
Bembidion varicolor (insect)	10	1 (10.0)	0.0022
Bembidion punctulatum (insect)	10	1 (10.0)	0.0012
Bembidion modestum (insect)	10	1 (10.0)	0.0005
Rhipicephalus sanguineus (insect)	39	3 (7.7)	0.0014
Calliphora terraenovae (insect)	31	2 (6.5)	0.0003
Calliphora (insect)	36	2 (5.6)	0.0427
Beetle metagenome (insect)	327	15 (4.6)	0.0022
Subpsaltria yangi (insect)	31	1 (3.2)	0.0011
Phormia (insect)	34	1 (2.9)	0.0082
Aedes albopictus (insect)	41	1 (2.4)	0.0002
Beta vulgaris (vegetable)	260	15 (5.8)	0.0046
Nepenthes rafflesiana (plant)	43	1 (2.3)	0.0006
Vitis vinifera (plant)	244	8 (3.3)	0.0021
Boechera stricta (plant)	1225	20 (1.6)	0.0002
Glechoma hederacea (plant)	88	1 (1.1)	0.0007
Rhizosphere metagenome	14155	172 (1.2)	0.0015
Cyprinus carpio (fish)	51	3 (5.9)	0.0003
Gorilla gorilla (mammal)	73	1 (1.4)	0.0009
Eye metagenome	64	1 (1.6)	0.0030
Aerosol metagenome	86	3 (3.5)	0.0661
Plastisphere metagenome	26	1 (3.8)	0.0005

Table S2. Genome relatedness between strain MW10^T and its closely related type strains of the genus *Dokdonella*

Taxa: 1, strain MW10^T (CP197094); 2, D. *immobilis* CGMCC 1.7659^T (FOVF00000000); 3, *D. koreensis* DS-123^T (CP015249).

		dDDH† value (%)		
		**1**	**2**	**3**
	**1**	–	21.0	21.1
ANI† value (%)	**2**	76.5	–	20.5
	**3**	76.6	75.2	–

†ANI, average nucleotide identity; dDDH, digital DNA-DNA hybridization.

Table S3. Comparison of cellular fatty acid compositions (%) of strain MW10^T and its closely related type strains of the genus *Dokdonella*

Taxa: 1, strain MW10^T; 2, *D. immobilis* JCM 15763^T; 3, *D. koreensis* KCTC 12396^T. All data were obtained from this study. Data are expressed as percentages of the total fatty acids, and fatty acids constituting less than 1.0% in all strains are not shown. Major components (>10.0%) are highlighted in bold. Symbols: tr, trace amount (<1.0%); –, not detected.

**Fatty acid**	**1**	**2**	**3**
Saturated:			
$C_{10:0}$	tr	tr	1.2
$C_{16:0}$	1.8	tr	4.8
Unsaturated:			
$C_{17:1}$ *ω5c*	–	–	1.2
Branched:			
iso-$C_{11:0}$	7.2	6.0	**11.5**
iso-$C_{14:0}$	tr	1.4	–
iso-$C_{15:0}$	**23.1**	**25.7**	9.7
anteiso-$C_{15:0}$	tr	tr	4.3
iso-$C_{16:0}$	**21.0**	**20.3**	6.4
iso-$C_{17:0}$	3.3	2.5	5.9
anteiso-$C_{17:0}$	tr	–	5.3
Hydroxy:			
iso-$C_{11:0}$ 3-OH	4.3	9.2	9.2
iso-$C_{12:0}$ 3-OH	tr	1.0	1.1
Summed feature*:			
3 ($C_{16:1}$ *ω7c* and/or $C_{16:1}$ *ω6c*)	5.6	2.4	2.1
9 (iso-$C_{17:1}$ *ω9c* and/or $C_{16:0}$ 10-methyl)	**23.8**	**23.8**	**29.3**

*Summed features are fatty acids that cannot be resolved reliably from another fatty acid using the chromatographic conditions chosen. The MIDI system groups these fatty acids together as one feature with a single percentage of the total.
